# Diagnostic high grade tuberculin sensitivity

**DOI:** 10.1016/j.idcr.2021.e01085

**Published:** 2021-03-26

**Authors:** Preetam Chappity, Deepika Mamidi, Sidharth Pradhan

**Affiliations:** a Department of ENT, All India Institute of Medical Sciences, Bhubaneswar, India; b ICMR Project, Department of ENT, All India Institute of Medical Sciences, Bhubaneswar, India

**Keywords:** Tuberculosis, Mantoux test, Skin test

A 31-year-old female presented with history of right sided cervical lymphadenopathy in level II, III and IV region for 4 months. The largest of them was 15 × 15 mm. The patient had no history of fever, weight loss, cough, malaise or any evident site of infection/lesion. She had received multiple courses of antimicrobials for persistent lymphadenopathy. The fine needle aspiration cytology, endoscopy, chest X ray, complete hematological evaluation and sputum evaluation did not yield any definitive diagnosis. A Mantoux test was performed to rule out an occult tubercular infection. 5 Tuberculin units of PPD (Purified protein derivative) was injected intra dermally in the left forearm. She developed significant induration (28 mm) and vesicles with necrosis by 48 h (Fig. 1). Although a positive test usually indicates an active or prior tubercular infection, this kind of acute hypersensitivity reaction strongly suggested the presence of an active tubercular infection [1]. She was started on empirical anti-tubercular treatment, with 4 drugs for 2 months and 3 drugs for the next 4 months. The patient had complete resolution of cervical lymphadenopathy and is disease free on 1 year follow up. Due to treatment with multiple antimicrobials prior to tuberculosis diagnosis, confirmatory display of acid fast bacilli is not possible in all cases. If Mantoux test displays a strong reaction with ulceration, it suggests an active ongoing infection and the patient can be empirically started on antitubercular treatment. Surgical approaches in tubercular lymphadenopathy can lead to sinus formation/delayed healing and should be avoided if possible.

**Fig. 1:**
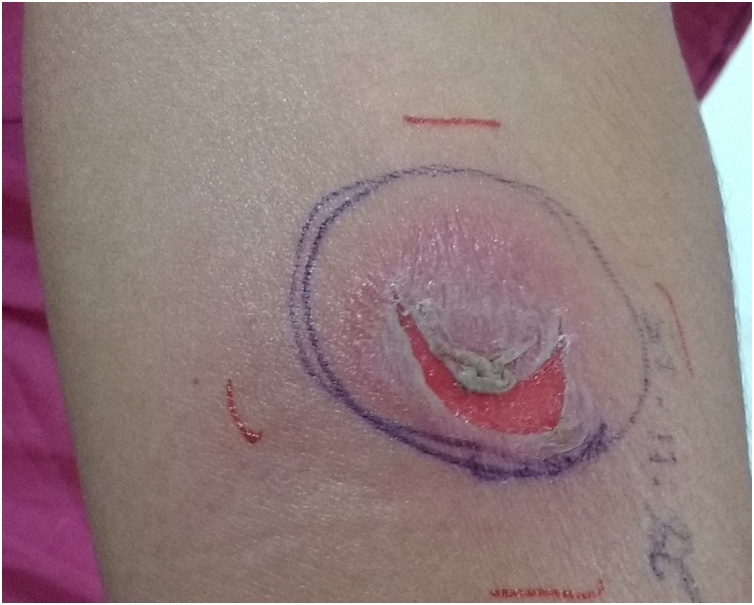
The test site after 48 h, showing induration with vesicles and necrosis.

## Funding

The study did not get any support from any funding organisation.

## Transparency document

Transparency document

## Declaration of Competing Interest

The authors report no declarations of interest.
